# Early Life Stress in Rodents: Animal Models of Illness or Resilience?

**DOI:** 10.3389/fnbeh.2018.00157

**Published:** 2018-07-31

**Authors:** Sahana Murthy, Elizabeth Gould

**Affiliations:** Princeton Neuroscience Institute, Princeton University, Princeton, NJ, United States

**Keywords:** early life stress, anxiety, depression, rodent models, resilience

Early life adversity is a known risk factor for mood and anxiety disorders in adult humans (Heim et al., 2010; Huh et al., 2014; Rehan et al., 2017). Given the prevalence of both maltreatment in childhood and mental illness in adulthood, understanding the neurobiological mechanisms of this connection is important as it may suggest targets for new therapeutic interventions. Ethical constraints on conducting studies with humans have highlighted the need for reliable and robust animal models that researchers can utilize to identify relevant neurobiological processes (Guzman et al., 2016). Since the work of Harlow and colleagues beginning in the 1940s, which involved raising infant macaques with cloth and wire mothers (reviewed in van der Horst and van der Veer, 2008), researchers have sought to develop useful animal models of early life adversity. These and other more recent studies have shown obvious behavioral abnormalities in monkeys subjected to early life stress (ELS) (Schino et al., 2001; Corcoran et al., 2012; Howell et al., 2014). Despite the relevance of these models to humans, nonhuman primates have practical and ethical limitations that are obstacles for their use in high-throughput studies. By contrast, animal models of early life stress in rodents, which were first used in the laboratory more than 50 years ago (Levine, 1957), have gained in usage.

One of the most commonly used manipulations to produce a rodent model of ELS has been maternal separation. Studies have shown that maternal separation in rats, as long as it is of sufficient duration (typically 3 h/day during the first 2 postnatal weeks of life) increases anxiety- and depressive-like behaviors in adulthood, suggesting that it has translational validity (Janus, 1987; Huot et al., 2001; Kalinichev et al., 2002; Romeo et al., 2003; Daniels et al., 2004; Lee et al., 2007; Wei et al., 2010; Masrour et al., 2018). However, other studies in both rats and mice have shown considerable variability in behavioral results from maternal separation, with several reports showing no behavioral effect (Lehmann et al., 1999; Eklund and Arborelius, 2006; Slotten et al., 2006; Millstein and Holmes, 2007; Savignac et al., 2011). In addition to inconsistent behavioral findings with this model, concerns have been raised about whether maternal separation mimics neglect, abuse or a combination of both. It has been reported that after prolonged separation, maternal behavior toward pups differs and these differences may be as important, if not more, than the lack of contact with the mother (Boccia and Pedersen, 2001; Huot et al., 2004). Some reports have also observed that dams increase maternal care post-separation possibly attenuating the effects of the separation itself (Millstein and Holmes, 2007). The type of human maltreatment that rodent maternal separation reflects might be important for establishing its translational validity, since human studies have separated early adverse experiences into several categories, including emotional abuse, emotional neglect, physical abuse, physical neglect and sexual abuse (Kendler et al., 2004; van Harmelen et al., 2010; Young and Widom, 2014; Rehan et al., 2017; Gallo et al., 2018) and some studies suggest that the type of maltreatment may be important for the adult outcome in terms of behavioral dysfunction (Huh et al., 2014; Young and Widom, 2014).

To address concerns about the unspecified nature of the maternal separation manipulation, researchers have developed another way to impair maternal care with the limited bedding/nesting model (Brunson et al., 2005; Cui et al., 2006; Ivy et al., 2008; Rice et al., 2008). The most extreme version of this model involves housing dams in a wire mesh floored cage with no bedding and a scarcity of material with which to make a nest, while variations involve just limiting nesting material (Walker et al., 2017). The result is an increase in maternal anxiety and fractured caregiving where behavior toward the pups might be interpreted as abusive (Rice et al., 2008). As with the earlier investigations of the maternal separation model, some studies using this manipulation reported evidence for increased anxiety- and depressive-like behavior in adulthood (Cui et al., 2006; Dalle Molle et al., 2012; Raineki et al., 2012; Wang et al., 2012), supporting its translational validity. However, other studies using this model failed to find an increase in anxiety- or depressive-like behavior (Brunson et al., 2005; Rice et al., 2008; van der Kooij et al., 2015; Johnson et al., 2018) raising questions about reliability similar to those observed with the maternal separation model.

Contradictory results of studies using both of these rodent models are puzzling and may be attributable to differences in experimental design. To fully understand these discrepancies, many factors must be considered (Figure 1). First, the genetic background of the experimental animal is important. Human studies have clearly shown genetic predisposition to mood and anxiety disorders and it follows that this factor should be considered in studies using experimental animals to model the human condition. Studies have shown varying effects of maternal separation on anxiety- and depressive-like behaviors in different strains of mice; the C57Bl/6 strain appears to be most resistant to stress compared to other strains, such as the Balb/c strain, which is inherently more anxious (Millstein and Holmes, 2007; Wei et al., 2010; Savignac et al., 2011). However, different studies using the same strain have reported conflicting results with seemingly identical ELS manipulations, so genetic strain differences cannot account for all of the variance in the literature. It should be noted, however, that individual subtler genetic differences within a specific rodent strain may be relevant to establishing vulnerability to such manipulations. That is, ELS manipulations likely impact some animals more than others and such variability may obscure overall group differences in behavior. Second, the sex of the animal should be considered. Somewhat paradoxically given that women exhibit greater prevalence of mood/anxiety disorders than do men (Altemus et al., 2014), several rodent studies have shown that ELS produces either no effect or a reduction in anxiety- and depressive-like behaviors in females (Lehmann et al., 1999; McIntosh et al., 1999; Eklund and Arborelius, 2006; Slotten et al., 2006). These unexpected results raise questions about whether the standard laboratory tests of anxiety- and depressive-like behavior, which were developed for use in males and typically involve measures of behavioral inhibition, are accurate measures of these states in female rodents, given known estrous cycle variations in behavioral activity levels. Clearly, the field would benefit from new sensitive behavioral assays that are useful for both sexes, particularly given the need to correct the under-emphasis of research on females (Clayton and Collins, 2014).

**Figure 1:**
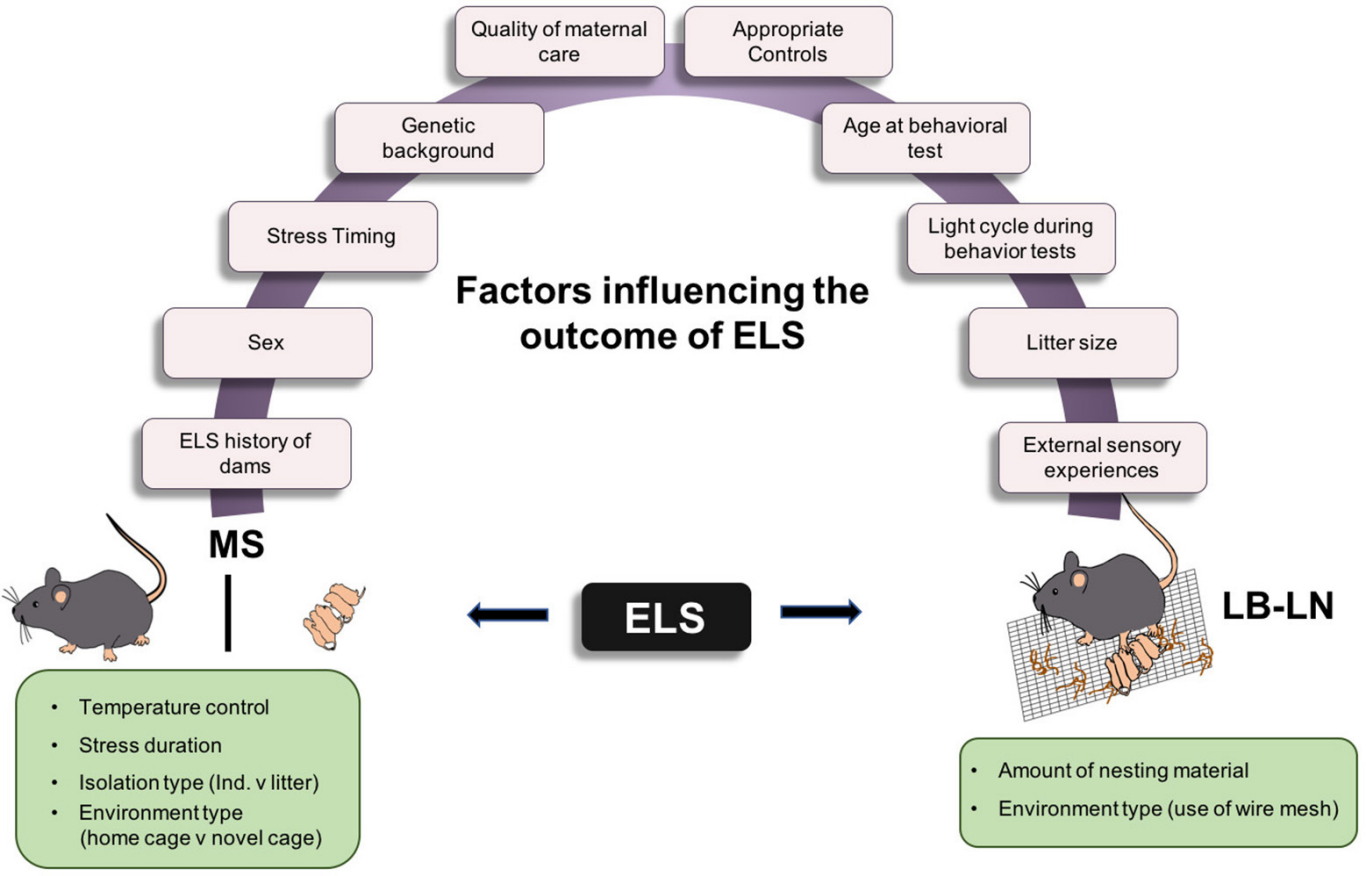
Factors influencing variability in ELS animal models. Cartoon depicting the two most commonly used models of ELS and the different factors influencing behavioral outcomes in adulthood. MS, Maternal separation stress; LB-LN, Limited bedding-Limited nesting.

The timing and duration of the stressful experience during the postnatal period may also be important to consider. In rodent studies, differential effects of early vs. late postnatal stress exposure on depressive-like behaviors have been demonstrated (van der Kooij et al., 2015; Peña et al., 2017). By contrast, however, a recent human study concluded that data on the link between childhood maltreatment and psychopathology do not fit a sensitive period theoretical model (Dunn et al., 2018), again raising questions about the direct translational validity of some ELS models in rodents. It is likely relevant that the HPA axis response to stress is attenuated in pups during the stress hyporesponsive period, a phenomenon that serves a protective effect on the developing brain (Sapolsky and Meaney, 1986). A similar state has been reported in humans up until about 1 year of age, but it does not extend throughout childhood when the majority of reported maltreatment occurs (Gunnar and Donzella, 2002).

The duration of stress seems to be more definitively associated with worse outcomes compared to the timing of stress, and data from human studies support a cumulative and/or recency model of stress effects on vulnerability to psychopathology (Dunn et al., 2018). To address the issue about the duration of stress as well as inconsistencies in the ELS literature, researchers have developed “two-hit” models that incorporate maternal separation followed by additional stress, either shortly thereafter or in adulthood. The models are based on the assumption that the first stressful period may create an internal vulnerability that is alone insufficient to manifest itself behaviorally, but when aggravated by subsequent stress, produces detectable behavioral changes. One set of such studies used longer periods of separation followed by early weaning of pups (George et al., 2010). Early weaning by itself has been shown to increase anxiety-like behavior in adulthood (Kikusui et al., 2004) and when combined with maternal separation, it not only increases anxiety-like behaviors but also results in hyperactivity, gene dysregulation and neuroanatomical changes to the brain; some of which have been observed in humans with a history of early life abuse. Another set of such studies used maternal separation and/or limited bedding followed by exposure to chronic stress in adulthood (Vargas et al., 2016; Peña et al., 2017). Both of these approaches mimic the “dose-response” or “cumulative” stress links to mental illness that have been described in humans. However, like the other rodent models of ELS, data from these two-hit models need to be interpreted with caution as null effects have also been reported (Santarelli et al., 2017; Tan et al., 2017).

Notwithstanding the potential importance of strain, sex, timing, duration, type of stress experience and other factors (Figure 1) across studies as reasons for variable results, it is clear that variable results can emerge even in the face of virtually identical experimental designs. What is the explanation for these differences? While we do not know for certain, there are some important points to consider. First, baseline housing and testing conditions may vary across laboratories in seemingly unspecified ways (Cavigelli et al., 2006; Sorge et al., 2014), adding additional stress to both control and experimental groups and potentially reducing the behavioral differences between them. Second, evidence suggests that rodent maternal behavior varies considerably even within control groups (Francis and Meaney, 1999). In other words, some rat and mouse dams may be more capable of compensating for the effects of maternal separation or limited bedding than others. This could be influenced by the early life experiences of the dams themselves and the amount of stress they were exposed to before entering breeding. This natural variation in maternal behavior may introduce additional variability into ELS-induced long-term behavioral outcomes. Third, perhaps related to the second point, rodent populations likely display considerable individual variability in response to ELS, such that depending on the cohort examined, statistically significant differences in anxiety- and depressive-like behavior may or may not be detectable. Thus, reproducible significant differences may require larger numbers of animals than are often used in such studies, consistent with what has been the norm for human studies (Collins and Tabak, 2014). In addition, these studies might be more informative if the data from rodents subjected to ELS manipulations were analyzed in ways that do not group them together with the assumption that they comprise a homogenous group. In searching for neurobiological mechanisms underlying behavioral signs of mental illness, it may be more fruitful to separate out the experimental animals that show robust ELS-induced increases in anxiety- and depressive-like behavior. This approach might reveal informative correlations between brain changes and relevant behaviors. While this suggestion makes experimental designs and statistical analyses more complicated than commonly used methods of comparing means between groups, it may produce more reliable results across laboratories.

Considering rodent populations as heterogeneous with regard to their susceptibility to ELS-induced behavioral changes would address an interesting parallel with humans. While the connection between early life adversity and mood/anxiety disorders in humans has been widely accepted, it is perhaps less well-known that the majority of people subjected to childhood maltreatment (>70%) do not show anxiety and depression symptoms that are clinically significant (Rehan et al., 2017). Thus, as with rodents, humans display a considerable amount of resilience and resistance to early life adversity, a phenomenon that deserves scientific attention as it may provide clues about how to encourage these characteristics in the entire population. Finally, it deserves mention that many people develop anxiety and mood disorders that are not retrospectively traceable to childhood maltreatment, so examining control rodents that score as more anxious/depressed despite a lack of prior stress manipulation may be informative as well. Here again, looking at individual differences within groups may be most informative and also help to reduce the inconsistency across studies using rodent models of stress-induced mental illness.

## Author contributions

SM wrote the article, edited the article and made the figure. EG wrote the article, edited the article and edited the figure.

### Conflict of interest statement

The authors declare that the research was conducted in the absence of any commercial or financial relationships that could be construed as a potential conflict of interest.

## References

[B1] Altemus M. Sarvaiya N. Neill Epperson C. (2014). Sex differences in anxiety and depression clinical perspectives. Front. Neuroendocrinol. 35, 320–330. 10.1016/j.yfrne.2014.05.004 24887405 PMC4890708

[B2] Boccia M. L. Pedersen C. A. (2001). Brief vs. long maternal separations in infancy: contrasting relationships with adult maternal behavior and lactation levels of aggression and anxiety. Psychoneuroendocrinology 26, 657–672. 10.1016/S0306-4530(01)00019-1 11500248

[B3] Brunson K. L. Kramár E. Lin B. Chen Y. Colgin L. L. Yanagihara T. K.. (2005). Mechanisms of late-onset cognitive decline after early-life stress. J. Neurosci. 25, 9328–9338. 10.1523/JNEUROSCI.2281-05.2005 16221841 PMC3100717

[B4] Cavigelli S. A. Guhad F. A. Ceballos R. M. Whetzel C. A. Nevalainen T. Lang C. M.. (2006). Fecal corticoid metabolites in aged male and female rats after husbandry-related disturbances in the colony room. J. Am. Assoc. Lab Anim. Sci. 45, 17–21. 10.1530/EC-17-0338 17089986

[B5] Clayton J. A. Collins F. S. (2014). Policy: NIH to balance sex in cell and animal studies. Nature 509, 282–283. 10.1038/509282a 24834516 PMC5101948

[B6] Collins F. S. Tabak L. A. (2014). Policy: NIH plans to enhance reproducibility. Nature 505, 612–613. 10.1038/505612a 24482835 PMC4058759

[B7] Corcoran C. A. Pierre P. J. Haddad T. Bice C. Suomi S. J. Grant K. A.. (2012). Long-term effects of differential early rearing in rhesus macaques: behavioral reactivity in adulthood. Dev. Psychobiol. 54, 546–555. 10.1002/dev.20613 22072233 PMC3298575

[B8] Cui M. Yang Y. Yang J. Zhang J. Han H. Ma W.. (2006). Enriched environment experience overcomes the memory deficits and depressive-like behavior induced by early life stress. Neurosci. Lett. 404, 208–212. 10.1016/j.neulet.2006.05.048 16790315

[B9] Dalle Molle R. Portella A. K. Goldani M. Z. Kapczinski F. P. Leistner-Segal S. Salum G. A.. (2012). Associations between parenting behavior and anxiety in a rodent model and a clinical sample: relationship to peripheral BDNF levels. Trans. Psychiatry 2:e195. 10.1038/tp.2012.126 23168995 PMC3565759

[B10] Daniels W. M. Pietersen C. Y. Carstens M. E. Stein D. J. (2004). Maternal separation in rats leads to anxiety-like behavior and a blunted ACTH response and altered neurotransmitter levels in response to a subsequent stressor. Metab. Brain Dis. 19, 3–14. 10.1023/B:MEBR.0000027412.19664.b3 15214501

[B11] Dunn E. C. Soare T. W. Raffeld M. R. Busso D. S. Crawford K. M. Davis K. A. (2018). What life course theoretical models best explain the relationship between exposure to childhood adversity and psychopathology symptoms: recency, accumulation, or sensitive periods? Psychol. Med. 26, 1–11. 10.1017/S0033291718000181 PMC6109629 29478418

[B12] Eklund M. B. Arborelius L. (2006). Twice daily long maternal separations in Wistar rats decreases anxiety-like behaviour in females but does not affect males. Behav. Brain Res. 172, 278–285. 10.1016/j.bbr.2006.05.015 16780968

[B13] Francis D. D. Meaney M. J. (1999). Maternal care and the development of stress responses. Curr. Opin. Neurobiol. 9, 128–134. 10.1016/S0959-4388(99)80016-6 10072372

[B14] Gallo E. A. G. Munhoz T. N. Loret de Mola C. Murray J. (2018). Gender differences in the effects of childhood maltreatment on adult depression and anxiety: a systematic review and meta-analysis. Child Abuse Negl. 79, 107–114. 10.1016/j.chiabu.2018.01.003 29428878

[B15] George E. D. Bordner K. A. Elwafi H. M. Simen A. A. (2010). Maternal separation with early weaning: a novel mouse model of early life neglect. BMC Neurosci. 11:123. 10.1186/1471-2202-11-123 20920223 PMC2955691

[B16] Gunnar M. R. Donzella B. (2002). Social regulation of the cortisol levels in early human development. Psychoneuroendocrinology 27, 199–220. 10.1016/S0306-4530(01)00045-2 11750779

[B17] Guzman D. B. Howell B. Sanchez M. (2016). Early life stress and development: preclinical science, in Posttraumatic Stress Disorder: From Neurobiology to Treatment, ed Bremner J. D. (Hoboken, NJ: Wiley-Blackwell), 61–80.

[B18] Heim C. Shugart M. Craighead W. E. Nemeroff C. B. (2010). Neurobiological and psychiatric consequences of child abuse and neglect. Dev. Psychobiol. 52, 671–690. 10.1002/dev.20494 20882586

[B19] Howell B. R. Grand A. P. McCormack K. M. Shi Y. LaPrarie J. L. Maestripieri D.. (2014). Early adverse experience increases emotional reactivity in juvenile rhesus macaques: relation to amygdala volume. Dev. Psychobiol. 56, 1735–1746. 10.1002/dev.21237 25196846 PMC4433484

[B20] Huh H. J. Kim S. Y. Yu J. J. Chae J. H. (2014). Childhood trauma and adult interpersonal relationship problems in patients with depression and anxiety disorders. Ann. Gen. Psychiatry 13:26. 10.1186/s12991-014-0026-y 25648979 PMC4304140

[B21] Huot R. L. Gonzalez M. E. Ladd C. O. Thrivikraman K. V. Plotsky P. M. (2004). Foster litters prevent hypothalamic-pituitary-adrenal axis sensitization mediated by neonatal maternal separation. Psychoneuroendocrinology 29, 279–289. 10.1016/S0306-4530(03)00028-3 14604606

[B22] Huot R. L. Thrivikraman K. V. Meaney M. J. Plotsky P. M. (2001). Development of adult ethanol preference and anxiety as a consequence of neonatal maternal separation in Long Evans rats and reversal with antidepressant treatment. Psychopharmacology 158, 366–373. 10.1007/s002130100701 11797057

[B23] Ivy A. S. Brunson K. L. Sandman C. Baram T. Z. (2008). Dysfunctional nurturing behavior in rat dams with limited access to nesting material: a clinically relevant model for early-life stress. Neuroscience 154, 1132–1142. 10.1016/j.neuroscience.2008.04.019 18501521 PMC2517119

[B24] Janus K. (1987). Effects of early separation of young rats from the mother on their open-field behavior. Physiol. Behav. 40, 711–715. 10.1016/0031-9384(87)90272-1 3671540

[B25] Johnson F. K. Delpech J. C. Thompson G. J. Wei L. Hao J. Herman P.. (2018). Amygdala hyper-connectivity in a mouse model of unpredictable early life stress. Transl. Psychiatry 8:49. 10.1038/s41398-018-0092-z 29463821 PMC5820270

[B26] Kalinichev M. Easterling K. W. Plotsky P. M. Holtzman S. G. (2002). Long-lasting changes in stress-induced corticosterone response and anxiety-like behaviors as a consequence of neonatal maternal separation in Long-Evans rats. Pharmacol. Biochem. Behav. 73, 131–140. 10.1016/S0091-3057(02)00781-5 12076732

[B27] Kendler K. S. Kuhn J. W. Prescott C. A. (2004). Childhood sexual abuse, stressful life events and risk for major depression in women. Psychol. Med. 34, 1475–1482. 10.1017/S003329170400265X 15724878

[B28] Kikusui T Takeuchi Y Mori Y. (2004). Early weaning induces anxiety and aggression in adult mice. Physiol. Behav. 81, 37–42. 10.1016/j.physbeh.2003.12.016 15059682

[B29] Lee J. H. Kim H. J. Kim J. G. Ryu V. Kim B. T. Kang D. W.. (2007). Depressive behaviors and decreased expression of serotonin reuptake transporter in rats that experienced neonatal maternal separation. Neurosci. Res. 58, 32–39. 10.1016/j.neures.2007.01.008 17298851

[B30] Lehmann J. Pryce C. R. Bettschen D. Feldon J. (1999). The maternal separation paradigm and adult emotionality and cognition in male and female Wistar rats. Pharmacol. Biochem. Behav. 64, 705–715. 10.1016/S0091-3057(99)00150-1 10593193

[B31] Levine S. (1957). Infantile experience and resistance to physiological stress. Science 126:405. 10.1126/science.126.3270.405 13467220

[B32] Masrour F. F. Peeri M. Azarbayjani M. A. Hosseini M. J. (2018). Voluntary exercise during adolescence mitigated negative the effects of maternal separation stress on the depressive-like behaviors of adult male rats: role of nmda receptors. Neurochem. Res. 43, 1067–1074. 10.1007/s11064-018-2519-6 29616445

[B33] McIntosh J. Anisman H. Merali Z. (1999). Short- and long-periods of neonatal maternal separation differentially affect anxiety and feeding in adult rats: gender-dependent effects. Dev. Brain Res. 113, 97–106. 10.1016/S0165-3806(99)00005-X 10064879

[B34] Millstein R. A. Holmes A. (2007). Effects of repeated maternal separation on anxiety- and depression-related phenotypes in different mouse strains. Neurosci. Biobehav. Rev. 31, 3–17. 10.1016/j.neubiorev.2006.05.003 16950513

[B35] Peña C. J. Kronman H. G. Walker D. M. Cates H. M. Bagot R. C. Purushothaman I.. (2017). Early life stress confers lifelong stress susceptibility in mice via ventral tegmental area OTX2. Science 356, 1185–1188. 10.1126/science.aan4491 28619944 PMC5539403

[B36] Raineki C. Cortes M. R. Belnoue L. Sullivan R. M. (2012). Effects of early-life abuse differ across development: infant social behavior deficits are followed by adolescent depressive-like behaviors mediated by the amygdala. J. Neurosci. 32, 7758–7765. 10.1523/JNEUROSCI.5843-11.2012 22649253 PMC3488459

[B37] Rehan W. Antfolk J. Johansson A. Jern P. Santtila P. (2017). Experiences of severe childhood maltreatment, depression, anxiety and alcohol abuse among adults in Finland. PLoS ONE 12:e0177252. 10.1371/journal.pone.0177252 28481912 PMC5421798

[B38] Rice C. J. Sandman C. A. Lenjavi M. R. Baram T. Z. (2008). A novel mouse model for acute and long-lasting consequences of early life stress. Endocrinology 149, 4892–4900. 10.1210/en.2008-0633 18566122 PMC2582918

[B39] Romeo R. D. Mueller A. Sisti H. M. Ogawa S. McEwen B. S. Brake W. G. (2003). Anxiety and fear behaviors in adult male and female C57BL/6 mice are modulated by maternal separation. Horm. Behav. 43, 561–567. 10.1016/S0018-506X(03)00063-1 12799173

[B40] Santarelli S. Zimmermann C. Kalideris G. Lesuis S. L. Arloth J. Uribe A.. (2017). An adverse early life environment can enhance stress resilience in adulthood. Psychoneuroendocrinology 78, 213–221. 10.1016/j.psyneuen.2017.01.021 28219813

[B41] Sapolsky R. M. Meaney M. J. (1986). Maturation of the adrenocortical stress response: neuroendocrine control mechanisms and the stress hyporesponsive period. Brain Res. 396, 64–76. 10.1016/0165-0173(86)90010-X 3011218

[B42] Savignac H. M. Dinan T. G. Cryan J. F. (2011). Resistance to early-life stress in mice: effects of genetic background and stress duration. Front. Behav. Neurosci. 5:13. 10.3389/fnbeh.2011.00013 21519375 PMC3075880

[B43] Schino G. Speranza L. Troisi A. (2001). Early maternal rejection and later social anxiety in juvenile and adult Japanese macaques. Dev. Psychobiol. 38, 186–190. 10.1002/dev.1012 11279595

[B44] Slotten H. A. Kalinichev M. Hagan J. J. Marsden C. A. Fone K. C. (2006). Long-lasting changes in behavioural and neuroendocrine indices in the rat following neonatal maternal separation: gender-dependent effects. Brain Res. 1097, 123–132. 10.1016/j.brainres.2006.04.066 16730678

[B45] Sorge R. E. Martin L. J. Isbester K. A. Sotocinal S. G. Rosen S. Tuttle A. H.. (2014). Olfactory exposure to males, including men, causes stress and related analgesia in rodents. Nat. Methods 11, 629–632. 10.1038/nmeth.2935 24776635

[B46] Tan S. Ho H. S. Song A. Y. Low J. Je H. S. (2017). Maternal separation does not produce a significant behavioral change in mice. Exp. Neurobiol. 26, 390–398. 10.5607/en.2017.26.6.390 29302206 PMC5746504

[B47] van der Horst F. C. van der Veer R. (2008). Loneliness in infancy: Harry Harlow, John Bowlby and issues of separation. Integr. Psychol. Behav. Sci. 42, 325–335. 10.1007/s12124-008-9071-x 18704609

[B48] van der Kooij M. A. Grosse J. Zanoletti O. Papilloud A. Sandi C. (2015). The effects of stress during early postnatal periods on behavior and hippocampal neuroplasticity markers in adult male mice. Neuroscience 311, 508–518. 10.1016/j.neuroscience.2015.10.058 26548415

[B49] van Harmelen A. L. de Jong P. J. Glashouwer K. A. Spinhoven P. Penninx B. W. Elzinga B. M. (2010). Child abuse and negative explicit and automatic self-associations: the cognitive scars of emotional maltreatment. Behav. Res. Ther. 48, 486–494. 10.1016/j.brat.2010.02.003 20303472

[B50] Vargas J. Junco M. Gomez C. Lajud N. (2016). Early life stress increases metabolic risk, hpa axis reactivity, and depressive-like behavior when combined with postweaning social isolation in rats. PLoS ONE 11:e0162665. 10.1371/journal.pone.0162665 27611197 PMC5017766

[B51] Walker C. D. Bath K. G. Joels M. Korosi A. Larauche M. Lucassen P. J.. (2017). Chronic early life stress induced by limited bedding and nesting (LBN) material in rodents: critical considerations of methodology, outcomes and translational potential. Stress 20, 421–448. 10.1080/10253890.2017.1343296 28617197 PMC5705407

[B52] Wang X. D. Labermaier C. Holsboer F. Wurst W. Deussing J. M. Muller M. B.. (2012). Early-life stress-induced anxiety-related behavior in adult mice partially requires forebrain corticotropin-releasing hormone receptor 1. Eur. J. Neurosci. 36, 2360–2367. 10.1111/j.1460-9568.2012.08148.x 22672268

[B53] Wei L. David A. Duman R. S. Anisman H. Kaffman A. (2010). Early life stress increases anxiety-like behavior in Balb c mice despite a compensatory increase in levels of postnatal maternal care. Horm. Behav. 57, 396–404. 10.1016/j.yhbeh.2010.01.007 20096699 PMC2849915

[B54] Young J. C. Widom C. S. (2014). Long-term effects of child abuse and neglect on emotion processing in adulthood. Child Abuse Negl. 38, 1369–1381. 10.1016/j.chiabu.2014.03.008 24747007 PMC4117717

